# SRC inhibition prevents P-cadherin mediated signaling and function in basal-like breast cancer cells

**DOI:** 10.1186/s12964-018-0286-2

**Published:** 2018-11-07

**Authors:** Ana Sofia Ribeiro, Ana Rita Nobre, Nuno Mendes, João Almeida, André Filipe Vieira, Bárbara Sousa, Filomena A. Carvalho, Joana Monteiro, António Polónia, Martina Fonseca, João Miguel Sanches, Nuno C. Santos, Raquel Seruca, Joana Paredes

**Affiliations:** 1 Epithelial Interactions in Cancer (EPIC), i3S - Instituto de Investigação e Inovação em Saúde, Universidade do Porto, Rua Alfredo Allen 208, 4200-135 Porto, Portugal; 2 Ipatimup, Institute of Molecular Pathology and Immunology of the University of Porto, Porto, Portugal; 3 ICBAS – Abel Salazar Biomedical Science Institute, Porto, Portugal; 4 Instituto de Medicina Molecular, Faculdade de Medicina, Universidade de Lisboa, Lisbon, Portugal; 5 FMUP, Medical Faculty of University of Porto, Porto, Portugal; 6 Institute for Systems and Robotics, Instituto Superior Técnico, Lisboa, Portugal

**Keywords:** P-cadherin, Dasatinib, Basal-like breast cancer, Src family kinase

## Abstract

**Background:**

Basal-like breast cancer (BLBC) is a poor prognosis subgroup of triple-negative carcinomas that still lack specific target therapies and accurate biomarkers for treatment selection. P-cadherin is frequently overexpressed in these tumors, promoting cell invasion, stem cell activity and tumorigenesis by the activation of Src-Family kinase (SRC) signaling. Therefore, our aim was to evaluate if the treatment of BLBC cells with dasatinib, the FDA approved SRC inhibitor, would impact on P-cadherin induced tumor aggressive behavior.

**Methods:**

P-cadherin and SRC expression was evaluated in a series of invasive Breast Cancer and contingency tables and chi-square tests were performed. Cell-cell adhesion measurements were performed by Atomic Force Microscopy, where frequency histograms and Gaussian curves were applied. 2D and 3D cell migration and invasion, proteases secretion and self-renew potential were evaluated in vitro. Student’s t-tests were used to determine statistically significant differences. The cadherin/catenin complex interactions were evaluated by in situ proximity-ligation assay, and statistically significant results were determined by using Mann-Whitney test with a Bonferroni correction. In vivo xenograft mouse models were used to evaluate the impact of dasatinib on tumor growth and survival. ANOVA test was used to evaluate the differences in tumor size, considering a confidence interval of 95%. Survival curves were estimated by the Kaplan-Meier’s method, using the log-rank test to assess significant differences for mice overall survival.

**Results:**

Our data demonstrated that P-cadherin overexpression is significantly associated with SRC activation in breast cancer cells, which was also validated in a large series of primary tumor samples. SRC activity suppression with dasatinib significantly prevented the in vitro functional effects of P-cadherin overexpressing cells, as well as their in vivo tumorigenic and metastatic ability, by increasing mice overall survival. Mechanistically, SRC inhibition affects P-cadherin downstream signaling, rescues the E-cadherin/p120-catenin complex to the cell membrane, recovering cell-cell adhesion function.

**Conclusions:**

In conclusion our findings show that targeting P-cadherin/SRC signaling and functional activity may open novel therapeutic opportunities for highly aggressive and poor prognostic basal-like breast cancer.

**Electronic supplementary material:**

The online version of this article (10.1186/s12964-018-0286-2) contains supplementary material, which is available to authorized users.

## Background

Basal-like breast cancer is an intrinsic molecular subtype (BLBC) [1, 2] associated with an aggressive biological behaviour and to a worse patient prognosis, which typically does not express hormonal receptors and HER2, constituting the vast majority of triple-negative breast carcinomas [3, 4]. Until now, targeted therapies are unavailable for these subgroup of breast cancer patients, mainly due to the absence of well-defined molecular targets, being chemo and radiotherapy the only therapeutic options to treat these patients. Consequently, it is urgent to find specific biomarkers/pathways that could impact the effective treatment of these poor prognosis breast carcinomas or accurate predictive molecular indicators that could be used to select patients for target therapies already available.

In the last years, our research team has been mainly focused in identifying key molecules that could be used as putative molecular targets in BLBC [5, 6]. We and others have found that the majority of these aggressive breast carcinomas express the basal marker P-cadherin (a cell-cell adhesion molecule), which overexpression is significantly associated to a worse disease-free and overall patient survival [5, 7, 8], as well as with a pro-invasive and stem-like cell behaviour [9–14]. Moreover, we demonstrated that P-cadherin inhibition decreases in vitro cell invasion and sensitizes breast cancer cells to radiation, as well as decreases in vivo tumorigenic ability, being indicated as a putative target for BLBC treatment [14]. We have also shown that P-cadherin-induced functional effects are due to the secretion of active proteolytic enzymes, such as MMPs, and of pro-invasive soluble cleaved-forms of P-cadherin [9], as well as to the activation of α6β4 integrin [13] and to the inhibition of the E-cadherin suppressive invasive function, by the disruption of the E-cadherin/p120ctn complex at the cell membrane [11]. It is well known that both cytoplasmic p120ctn and α6β4 integrin activation can induce the phosphorylation of Src Family Kinases (SRC) and focal adhesion kinase (FAK), as well as the activation of Rac1 small GTPase [15–17]. There are several lines of evidence showing that tyrosine phosphorylation of these enzymes may play a role in the disruption of cell-cell adhesion, due to loss of cadherin/catenin association, with a huge impact in cancer progression. Importantly, we have described that P-cadherin expression induces SRC activation in breast cancer cells, suggesting that SRC signaling is being upstream regulated by this cell-cell adhesion molecule [13, 18]. Thus, in order to evaluate the role of P-cadherin/SRC signaling activation in BLBC cells, we have used dasatinib (an FDA approved SRC inhibitor) to block this pathway and prevent P-cadherin induced aggressive behavior of BLBC cells.

## Methods

### Cell culture and siRNA transfections

Human breast cancer cell (BCC) lines were obtained as follows: MCF-7/AZ was provided by Prof. Marc Mareel (Ghent University, Belgium); SUM-149 was kindly provided by Dr. Stephen Ethier (University of Michigan, USA); BT20 and MDA-MB-468 were obtained from American Type Culture Collection (Manassas, VA, USA). The MCF-7/AZ cell line is a variant of the human BCC line MCF-7 that expresses basal levels of P-cadherin. This cell line was retrovirally transduced to encode only EGFP (LZRS-IRES-EGFP plasmid, MCF-7/AZ.Mock cell line) or both P-cadherin and EGFP (LZRS-P-cad-IRES-EGFP plasmid, MCF-7/AZ.P-cad cell line), as previously described [19]. MCF-7/AZ.Mock cell line was used as a control.

Cells were routinely maintained at 37 °C, 5% CO_2_, in the following media (Invitrogen Ltd., Paisley, UK): 50% DMEM + 50% HamF12 (MCF-7/AZ, SUM149) and DMEM (BT20, MDA-MB-468), supplemented with 10% heat-inactivated fetal bovine serum (FBS), 100 IU/ml penicillin and 100 mg/ml streptomycin (Invitrogen, UK). SUM149 medium was supplemented with 5 μg/ml of insulin and 1 μg/ml of hydrocortisone (Sigma-Aldrich, St. Louis, MO, USA).

Transfections of validated small interfering RNA (siRNA) specific for P-cadherin (Hs_CDH3_6, GW Validated siRNA, Qiagen, Cambridge, MA, USA) and SRC (Hs_SRC_1 FlexiTube and Hs_SRC_11 FlexiTube siRNA, Qiagen) was carried out using Lipofectamine 2000 (Invitrogen), in a final concentration of 50 nM (CDH3 and SRC1) and 100 nM (SRC11), according to the manufacturer’s recommended procedures. After incubation for 5 min, the siRNA and Lipofectamine 2000 solutions were mixed, incubated for 20 min, and added to cell culture medium. A siRNA control, with no homology to any gene, was also used (Qiagen), to verify the specificity of the results obtained.

### Drugs and antibodies

Dasatinib was obtained from Sequoia Research Products (Pangbourne, UK), dissolved in DMSO, and cells were treated at a final concentration of 100 nM each 24 h for a total period of 48 h. PP2 (P0042, Sigma-Aldrich) was dissolved in DMSO and added to the cells at a final concentration of 1 and 10 μg/ml.

Monoclonal antibodies were obtained as follows: E-cadherin (WB) (Takara Bio Inc., Shiga, Japan); P-cadherin (WB) and p120ctn (BD Transduction Laboratories, Palo Alto, CA, USA); E-cadherin (PLA, IF, IHQ), P-cadherin (IF), total Src (L4A1) and pSRC(Tyr416) (Cell Signaling Technology); β-actin (Santa Cruz Biotechnologies, Santa Cruz, CA, USA). The detailed conditions for each antibody are described on Additional file 1: Table S1.

### Cell-cell adhesion measurements by atomic force microscopy

Cells were cultured in a tissue culture dish at a low cell density concentration to have only dispersed cells, without reaching cell confluence. On the day of the experiment, cells were washed twice with PBS and 1 mL of serum-free DMEM was added. For the cell-cell adhesion experiments, tipless arrow TL1 cantilevers (Nanoworld, Neuchatel, Switzerland) were used, with a nominal spring constant of 0.03 N/m. Cantilevers were cleaned for 15 min with UV light and coated with poly-D-lysine (50 μg/ml) for at least 30 min. Cantilevers were left in the poly-D-lysine solution until used.

Isolated cell suspensions were obtained after 1 h of incubation in PBS (without trypsin treatment) of confluent cells in a separate dish and subsequent pipette dispersion. The cells and the functionalized cantilevers were mounted on the CellHesion module (JPK Instruments, Berlin, Germany), with a 100-μm z-range piezoelectric scanner, connected to the NanoWizard II atomic force miscroscope mounted on the top of an Axiovert 200 inverted microscope. 100 μL of cells suspension were injected into the cells substrate dish. Cells were allowed to settle for 30 s before capturing by a functionalized cantilever of one cell using the AFM contact mode. After 30 s of cantilever pressing onto the cell, the cantilever was raised 100 μm on the z-range and the attached cell allowed to rest for 1 min before initiating the contact with an adherent cell on the substrate underneath. Cell-cell contact was established with an applied force of 300 pN, in constant height and closed-loop mode. The AFM tip resonant frequency was maintained at 2 Hz and a cell-cell contact time of 5 s was maintained before cantilever retraction, with a z-range displacement of 50 μm). Five force-distance curves were performed on each cell on the substrate, with a 5 s pause between them. A maximum of 8 different adherent cells on the substrate were tested with the same cell attached to the AFM cantilever.

### Western blot

Protein lysates were prepared from cultured cells in catenin lysis buffer [1% (v/v) Triton X-100 and 1% (v/v) NP-40 (Sigma) in desionized PBS] with 1:7 proteases inhibitors cocktail (Roche Diagnostics GmbH). Cells were washed twice in PBS and were allowed to lyse in 500 μl of catenin lysis buffer for 10 min, at 4 °C. Lysates were then submitted to vortex 3 times, centrifuged 10 min (14,000 rpm) and supernatants were collected. Protein concentration was determined using a Bradford assay (BioRad protein quantification system, BioRad, Hercules, CA, USA). Proteins were dissolved in Sample Buffer [Laemmli with 5% (v/v) 2-β-mercaptoethanol and 5% (v/v) bromophenol blue] and boiled for 5 min at 95 °C. Samples were then separated by 8% SDS-PAGE. Proteins were transferred into nitrocellulose membranes (Amersham Hybond ECL, Amersham biosciences, Buckinghamshire, UK) at 130 V for 1 h. For immunostaining, membranes were blocked with 5% (w/v) non-fat dry milk in PBS containing 0.5% (v/v) Tween-20. Membranes were subsequently incubated with primary antibodies for 1 h at RT (E-cadherin – dilution 1:1000; P-cadherin – dilution 1:500; p120ctn – dilution 1:1000; tSrc – dilution 1:500; pSRC(Tyr416) – dilution 1:500 and β-actin – dilution 1:1000), followed by four 5 min washes in PBS/Tween-20 and incubation with horseradish peroxidase-conjugated secondary antibodies (donkey anti-goat, goat anti-mouse, goat anti-rabbit – dilution 1:2000) for 1 h. Membranes were then washed for 30 min in PBS/Tween-20. Proteins were detected using ECL reagent (Amersham biosciences) as a substrate. Blots were exposed to an autoradiographic film. Band quantification of western blots was performed using Quantity One (BioRad). Each immunoblot was performed at least three times, and the ones selected for figures are representative experiments.

### 3D breast cancer cells spheroids culture

Aggregoids were obtained by performing the slow aggregation assay, as previously described [19]. Briefly, 1 × 10^4^ cells in 200 μL medium were seeded on solidified agar in a 96-well plate for 12 h. Medium containing the formed cellular aggregates (10–100 cells) were centrifuged and further embedded in acid solubilized rat tail collagen I gels (1.7 mg/ml collagen I in PBS; 22.5 mM NaHCO_3_; 8 mM NaOH; 9% of non-supplemented culture medium). Cellular aggregoids were plated in a 8-well coverslip bottom chambers (IBIDI, Germany) followed by 30 min incubation at 37 °C to allow polymerization. After that time, medium with or without dasatinib was added to the cellular 3D aggregoid cultures. The in vitro behaviour was followed by time-lapse microscopy (Leica microscope - DMI6000B with Adaptative Focus Control) during 18 h. Further, the images taken were converted into a time-lapse movie, and quantitative analysis of the number and length of protrusive structures was performed using Leica AF6000 software.

### Immunofluorescence analysis

Cells were cultured on glass coverslips, fixed with 4% paraformaldehyde (20 min), treated with NH_4_Cl (50 mM) for 10 min, washed with PBS, and permeablilized with 0.1% Triton X-100 in PBS for 5 min, at room temperature. Non-specific binding was blocked by treatment with 5% BSA in PBS, for 30 min at room temperature. Cells were then stained Orange-phalloidin (Sigma, St. Louis, MO, USA) in order to visualize F-actin, for 30 min, or with the monoclonal mouse antibody for p120ctn (dilution 1:100) and monoclonal rabbit antibody for E-cadherin (dilution 1:100) for 1 h, followed by conjugated goat anti-mouse and anti-rabbit secondary IgG (Dako Cytomation, Carpinteria, CA, USA), for 1 h at room temperature. After another wash with PBS, each sample was mounted with Vectashield (Vector Laboratories, Inc., Burlingame, CA, USA) containing 4,6-diamidine-2-phenylindolendihydrochrolide (DAPI) and visualized with Leica DM 2000 microscope (Leica).

### Matrigel invasion assays

Matrigel invasion assay was performed using 8 μm pore size BD BioCoat™ Matrigel Invasion Chambers (BD Biosciences), according to manufacturer’s recommendations. Briefly, 24 h after siRNA transfection, 5 × 10^4^ cells were added to the upper compartment of the chamber, with or without dasatinib treatment. In the lower compartment, only fresh medium supplemented with 10% FBS was added. After 24 h (BT20) or 48 h (MCF-7/AZ) of incubation at 37 °C, the upper surface of the filter was cleared from non-invasive cells with a cotton swab and washed with PBS. The invasive cells were fixed with cold methanol and stained with DAPI. Invasive cells were scored by counting the cells in the filter with a fluorescence microscope (Zeiss Imager Z1), at 100× of magnification. Invasion index expresses the ratio between invasive cells from the different conditions relative to the control cells.

### Wound-healing assay

After transfection, for 24 h, with the respective siRNAs, cells were tripsinized and seeded for another 24 h, to allow the cells to form a complete monolayer. After they became confluent, wounds were carefully introduced across the cell monolayer, so that the surrounding cells were not disturbed. The medium was replaced with fresh complete medium, with and without dasatinib treatment. Pictures were taken at 100× of magnification in an inverted microscope (Zeiss), and measurements of the distance between the wound edges were made at specific time points.

### Zymography

The conditioned medium collected from the several cell cultures, which were grown 6-well plates coated with collagen type I, was analysed for proteinases activity using β-casein zymography. Samples were mixed with sample buffer [0.03% bromophenol blue, 0.25 M Tris-HCl pH 6.8, 10% SDS (*w*/*v*) and 4% sucrose (w/v)] and electrophoresed, under non-reducing conditions, on 10% polyacrilamide gels containing 0.1% (w/v) β-casein from bovine milk (Sigma). After electrophoresis, gels were washed twice, for 30 min, in 2% (v/v) Triton X-100 (Sigma) at room temperature, in order to remove SDS. β-casein gels were then incubated in a substrate reaction buffer [0.2 M NaCl, 5 mM CaCl_2_, 1% (v/v) Triton X-100 in 50 mM Tris-HCl, pH 7.4], during 72 h, and finally stained with Coomassie Blue Staining Solution [0.1% (w/v) Coomassie Blue R250 in 10% (v/v) acetic acid and 40% (v/v) methanol], for 25 min. The gels destaining was performed in a solution with 20% methanol and 10% acetic acid, until bands start to become visible. Enzymatic activity was visualized as a clear band against the blue background of stained casein gels, and MMPs were identified by their molecular weight. Quantification of band density was carried out using the Quantity One software (version 4.0, BioRad, Hercules, CA, USA).

### In situ proximity ligation assay (PLA)

Cells were deposited on glass slides and fixed with methanol for 10 min. PLA was performed using the Duolink kit (Olink Bioscience, Sweden), according to manufacturer’s recommendations. The following combination of primary anti-human antibodies were used against: P-cadherin (dilution 1:50, rabbit polyclonal IgG, Cell Signaling), E-cadherin (dilution 1:100, mouse monoclonal IgG1, clone HECD-1, Takara Bio Inc., Shiga, Japan; or dilution 1:50, rabbit monoclonal IgG, Cell Signaling), p120ctn (dilution 1:100, mouse monoclonal IgG1, clone 98, BD Biosciences). The nuclei were counterstained with DAPI. Slides were analyzed with fluorescence microscopy (Zeiss Imager Z1 microscope) for visualization of bright fluorescent red signals consistent with protein-protein interaction events. Ten stacks per image were taken and a minimum of five fields per experiment were evaluated. The Blobfinder V3.2 free software (Centre for Image Analysis, Uppsala, Sweden) was used to quantify the number of blobs (or dots) present in each condition. For cell culture experiments, an average of blobs/cells of at least three independent experiments was performed and was normalized to the control condition. For paraffin embedded tumors, the specific number of blobs/cell was analysed. The respective negative controls were used in each experiment.

### Internuclear profile analysis

A software application was developed in order to generate and analyze the internuclear profile of proteins. In each immunofluorescent image, pairs of cells were selected for analysis in a semi-automated manner. The mapping and quantification of the protein expression level was performed by computing, respectively, 1D IN and RD intensity profiles of two contiguous cells and within one single cell. To cope with cell size and shape variability, a geometric compensation algorithm was developed in a Bayesian framework. The method was designed as an iterative algorithm composed by the following steps: (i) profile extraction from selected single cells (in case of RD) or pairs of cells (in case of IN); (ii) image map building by stacking fluorescence profiles together in columns after length normalization; (iii) denoising of map image (in which multiplicative noise described by a Poisson distribution is assumed); (iv) geometric compensation of each 1D column profile minimizing the overall variability of the map along the lines (horizontal direction); and (v) computation of the average and standard deviation profiles using the compensated map. After the extraction of the data, the maximum mean ratio (MMR) parameter was calculated dividing the maximum fluorescence value (numerator) by the fluorescence mean (denominator).

### Animal studies

N:NIH(S)II-nu/nu mice, strain produced by Ipatimup, were housed, breeded and maintained at the CIM-FMUP Animal House, in a pathogen-free environment under controlled conditions of light and humidity.The experiments consisted on the orthotopic injection in the mammary fat pad of female mice, with 6–8 weeks of age, with 2 × 10^6^ cells from each P-cadherin-overexpressing breast cancer models (SUM149PT, *n* = 17; BT20, *n* = 15; MDA-MB-468, *n* = 13) using a 25G needle. When the induced primary tumours reached a median volume of 100 mm^3^, mice were randomized into two groups, and started the oral daily treatment with dasatinib, whereas the others were treated just with the drug vehicle, until the end of the experiment. Doses of dasatinib to be administered were prepared weekly by dilution in citrate buffer 80 mM (pH = 3.1). The citrate buffer was prepared as follows: sodium citrate powder was diluted in citric acid solution 1 M, obtained by dissolving citric acid in deionized water, to a final concentration of 80 nM and with pH = 3.1. The citrate buffer solution was made every 3 weeks, filtered in flow chamber and kept at 4 °C. Every week, daily doses of dasatinib were prepared to a final concentration of 10 mg/kg, in order to treat mice of both experimental models. Mice (6–8 per group) were weighted, and tumor width and length were measured with calipers, at least, once a week. Tumor volume was estimated by using the equation, V = 0.5 × a × b^2^, where V is the volume, a is the length of the major axis of the tumor, and b is the length of its minor axis. Tumors were surgically removed when an average volume of 1000 mm^3^ was reached as previously described. Mice were maintained to evaluate the impact of dasatinib in mice overall survival, for a maximum of 210 days, unless they showed any signs of disease progression. For that, mice were euthanized accordingly to the established Endpoints. Upon extraction, Primary tumors were fixed in 10% buffered formalin and then embedded in paraffin, sectioned, and stained with haematoxylin and eosin. Immunohistochemistry for E- and P-cadherin was performed in tumors sections.

### Immunohistochemistry

A series of 467 primary invasive breast carcinomas were retrieved from the Pathology Department, Hospital Xeral-Cíes, Vigo, Spain, diagnosed between 1978 and 1992. The tumors have been characterized for clinical and pathological features as previously described [11].

IHC was performed as previously described [6]. The membrane staining extension was evaluated on a scale of 0 to 3 (0–0% to 10%; 1–10% to 25%; 2–25% to 50%; 3–50% to 100%). For E-cadherin and total Src, scores of 0 and 1 were considered negative and immunoreactivity of 2 and 3 was scored as positive. For P-cadherin all tumors presenting an unequivocal membranous staining, in at least 10% of the neoplastic cells (≥1), were scored as positive. For pSRC(Tyr416) tumors presenting membrane or cytoplasmic staining, in at least 10% of the neoplastic cells (≥1), were scored as positive. Some samples could not be assessed due to core falling or lack of enough biological material to analyze.

### Statistical analyses

All other statistical analyses were performed by Graph Pad Prism version 5.0c software (Graph Pad Software, San Diego, CA, USA), unless otherwise stated. *P* values less than 0.05 were considered statistically significant.

For the AFM analysis, Student’s t-tests were used to determine statistically significant differences. Frequency histograms were performed in Origin (OriginLab, Northampton, MA, USA) and Gaussian curves were applied.

Quantitative parameters of Internuclear profiles (normalized to a constant length of 100 arbitrary units) in P-cadherin cells were analyzed using a Mann-Whitney test with a Bonferroni correction.

For the in vivo xenograft assays, ANOVA test was used to evaluate the differences in tumor size, considering a confidence interval of 95%. Survival curves were estimated by the Kaplan-Meier’s method, using the log-rank test to assess significant differences for mice overall survival.

Concerning the functional in vitro assays, all were performed independently and in triplicate.

For statistical analysis of the immunohistochemistry results, contingency tables and chi-square tests were performed by SPSS 15.0 software package for Windows (SPSS, Inc., USA), to estimate the relationship between staining patterns of P-cadherin and pSRC (Tyr416).

All statistical tests were two-sided.

## Results

### P-cadherin/*CDH3* overexpression is significantly associated with SRC activation in human breast cancer cells

**Fig. 1:**
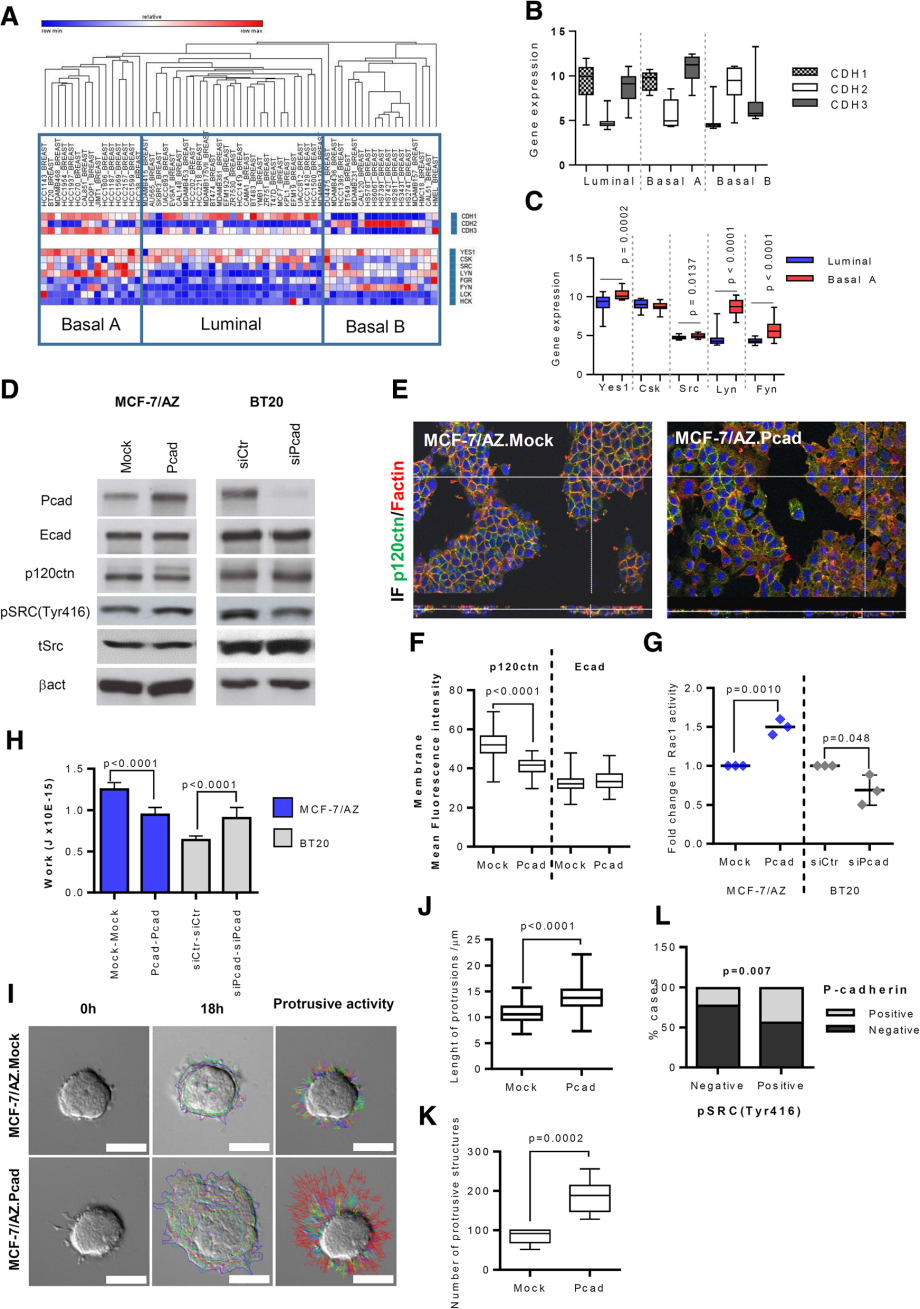
P-cadherin overexpression associates with SRC activation correlating with an aggressive behavior. **a** Gene expression profile of SRC family members from BCC lines retrieved from the CCLE database [20]. Unsupervised hierarchical clustering based on classical cadherins expression (*CDH1*, *CDH2*, *CDH3*), subdivide the BCC lines into their molecular subtype (Luminal, Basal A and Basal B). **b** Box-plot representation of *CDH1*, *CDH2* and *CDH3* gene expression values in the different breast cancer molecular subtypes. **c** Box-plot for SRC associated genes expression in the Luminal and Basal A BCC subtypes. *P*-values indicate significantly differences observed in gene expression profile from Luminal and Basal A BCC lines. **d** Western blot analysis of P-cadherin, E-cadherin, p120ctn, pSRC(Tyr416), total SRC and β-actin proteins after P-cadherin overexpression and P-cadherin silencing, respectively. **e** Dual immunofluorescence confocal images using an anti-p120ctn antibody (green), F-actin (red) and DAPI (blue). **f** Internuclear profile for p120ctn and E-cadherin, obtained through the computational analysis of immunofluorescence images from MCF-7/AZ BCC model. *P*-value indicate significant differences observed in the membrane mean fluorescent intensity for p120ctn between MCF-7/AZ.Mock versus MCF-7/AZ.Pcad cells. **g** Quantification of the active Rac1-GTP, measured by a G-Lisa assay after P-cadherin overexpression and P-cadherin silencing, respectively. **h** Average values of Work (J), representing the cell-cell adhesion strength, using AFM Force Spectroscopy analysis. Student’s t-test was used to determine statistically significant differences, and P-values are indicated in the figure. **i** Representative Images of time-lapse microscopy for BCC spheroids embedded in collagen type I. Scale bar = 50 μm. **j** Box-plot quantification of the extension (μm) of invasive protrusions. **k** Box-plot quantification of the number of invasive protrusions. **l** Representation of the percentage of cases positive for both pSRC(Tyr416) and P-cadherin expression. *P*-value was calculated by Chi-square test in order to estimate the relationship between staining patterns of P-cadherin and pSRC(Tyr416)

In order to confirm the association between P-cadherin and SRC activation, we started by analysing the expression of *CDH3* (P-cadherin codifying gene) and SRC associated genes (*SRC, YES1, FYN, LYN, LCK, CSK*) in a public database of breast cancer cell lines belonging to different molecular subtypes (Fig. 1a) [CCLE database [20]]. Interestingly, cell lines showing the highest levels of *CDH3* also present a significantly increased expression of *YES, SRC, LYN* and *FYN*. Accordingly, most of these cell lines were classified as Basal A, which are the ones that better resemble the BLBC subtype (Fig. 1b-c).

We then validated the association between P-cadherin and SRC activation using two established in vitro breast cancer cell models, where P-cadherin expression was manipulated. P-cadherin overexpressing cells showed increased levels of pSRC(Tyr416), while P-cadherin silencing led to a significant decrease of pSRC(Tyr416) levels (Fig. 1d and Additional file 1: Figure S1A and B). No alterations in the total levels of SRC were found by Western blot. E-cadherin and p120ctn were also evaluated in both models and the level of expression and localization of both proteins were analysed by the fluorescence internuclear profiles [21]. In contrast to what was observed for E-cadherin expression, which did not alter upon genetic manipulation of P-cadherin, a decrease of p120ctn membrane intensity was observed in P-cadherin overexpressing cells (Fig. 1e-f). Moreover, a significant increase in Rac1 activity, a downstream target of cytoplasmic p120ctn, was observed in cells with P-cadherin overexpression, as well as the silencing of P-cadherin in BT20 cells was accompanied by a significant decrease in Rac1 activation (Fig. 1g). Cell-cell adhesion strength was also clearly compromised in P-cadherin overexpressing cells, as demonstrated by atomic force microscopy (AFM) measurements (Fig. 1h), as well as a significant increase in the protrusive and invasive behavior (Fig. 1i-k and Additional file 1: Figure S1C) of breast cancer cells was promoted, by the evaluation of 3D spheroids embedded in collagen type I matrix by time-lapse microscopy.

These findings, together with the fact that P-cadherin affects the E-cadherin/p120ctn complex membrane stabilization, and that its juxtamembrane domain is crucial for its pro-invasive function [11], led us to presume that P-cadherin could be modifying cell-cell adhesion and invasion capacity through p120ctn delocalization occurring downstream of SRC activation signaling.

Finally, to validate in vivo the association between pSRC(Tyr416) and P-cadherin, a series of 416 primary breast tumors was studied by immunohistochemistry (Table 1). A significant association was found between the presence of both proteins, where 44% (18 in 41 cases) of the tumors with pSRC(Tyr416) were also positive for P-cadherin (Fig. 1l).

**Table 1:** Characterization of the tumor series, conserning tumor pathological features and molecular markers

		N	%
Histological grade	1	75	18.0
	2	117	28.1
	3	204	49.0
	Missing	20	4.8
ERα	Negative	140	33.7
	Positive	274	65.9
	Missing	2	0.5
HER2	Negative	352	84.6
	Positive	60	14.4
	Missing	4	1.0
EGFR	Negative	395	95.0
	Postive	21	5.0
	Missing	0	0.0
CKS	Negative	357	85.8
	Positive	59	14.2
	Missing	0	0.0
CK14	Negative	394	94.7
	Postive	22	5.3
	Missing	0	0.0
Vimentin	Negative	345	82.9
	Postive	69	16.6
	Missing	2	0.5
CD44	Negative	205	49.3
	Positive	210	50.5
	Missing	1	0.2
Subtype	Luminal	303	72.8
	Her2	29	7.0
	Triple-Negative	84	20.2
	Missing	0	0.0

### SRC activity suppression with dasatinib inhibits the in vitro functional activity induced by P-cadherin overexpression

Taking into account the results showing a positive association between P-cadherin expression and SRC activation, we speculated if the downstream SRC signalling could be an effective target for poor prognostic P-cadherin-overexpressing breast cancer. This strategy would overcome the lack of FDA-approved drugs directly targeting P-cadherin.

**Fig. 2:**
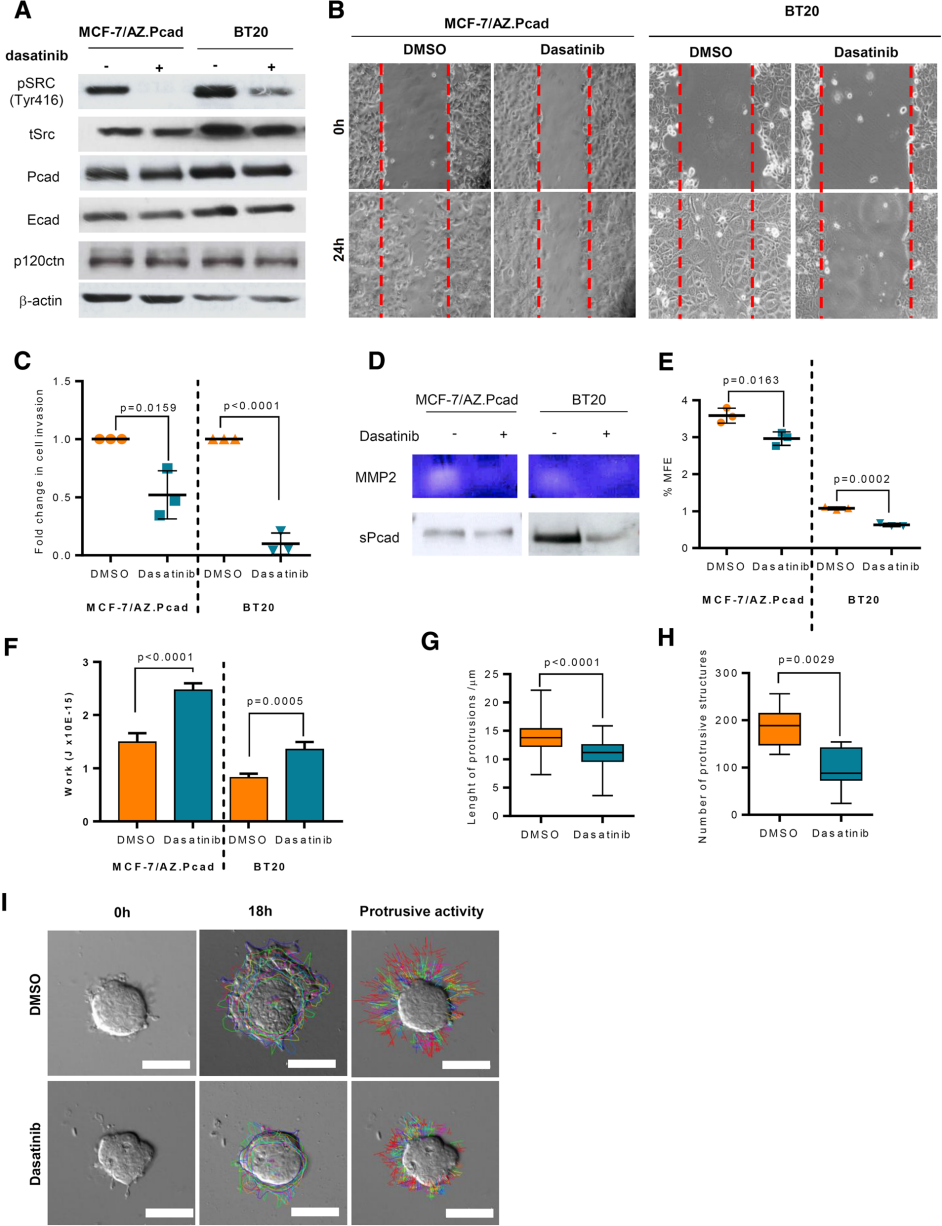
Dasatinib treatment inhibits the in vitro functional activity induced by P-cadherin expression. **a** Western blotting for pSRC(Tyr416), total Src, P-cadherin, E-cadherin and p120ctn in P-cadherin overexpressing cells after dasatinib treatment (100 nM) for 48 h. Protein levels of β-actin were analyzed and used as the loading control. **b** Representative experiment from a wound healing migration assay, in both P-cadherin overexpressing BCC (MCF-7/AZ.Pcad and BT20), treated with 100 nM of dasatinib or DMSO for 24 h. **c** Fold change in the number of invasive cells, evaluated by the matrigel invasion assay for both MCF-7/AZ.Pcad and BT20 treated with DMSO or 100 nM of dasatinib. **d** Zymography for MMP2 activity and Western blotting for sP-cad, using the conditioned medium from cells treated with DMSO or 100 nM of dasatinib for 48 h, in both MCF-7/AZ.Pcad and BT20 BCC models. **e** Mammosphere forming assay was performed for both cell models. The *P*-values indicate the statistically significant difference between DMSO and dasatinib treated cells, in both P-cadherin BCC models. **f** Average values of Work (J), representing the cell-cell adhesion strength, of both BCC cancer cell models treated with DMSO and dasatinib, using AFM Force Spectroscopy analysis. **g** Box-plot quantification of the length (μm) and **h** number of invasive protrusions of MCF-7/AZ.Pcad spheroids, treated with DMSO or 100 nM dasatinib, for 24 h. *P*-values < 0.05 were considered statistically significant. **i** Representative Images of time-lapse microscopy for BCC spheroids embedded in collagen type I, for MCF-7/AZ.Pcad treated with DMSO and dasatinib. Scale bar = 50 μm

Thus, we examined the effect of SRC inhibition in the previously defined in vitro functional activity induced by P-cadherin expression, by treating cells with 100 nM of dasatinib (concentration that does not affect the metabolic state and viability of breast cancer cells – Additional file 1: Figure S1D). Breast cancer cells treated with dasatinib showed a decrease of pSRC(Tyr416) expression (Fig. 2a and Additional file 1: Figure S1E); moreover, this treatment led to a decrease in cell migration (Fig. 2b) and cell invasion (Fig. 2c), associated with a decrease of MMP2 activity and sP-cad expression in the conditioned media (Fig. 2d), as well as with a decreased mammosphere forming efficiency (MFE) (Fig. 2e).

Additionally, cell-cell adhesion was highly promoted in P-cadherin overexpressing cells treated with dasatinib, as observed by the brightfield images and quantified by AFM in both cell lines after SRC inhibition (Fig. 2f). Additionally, when we evaluated the in vitro 3D invasion ability of P-cadherin overexpressing cells after dasatinib treatment, we observed a significant decrease in the number and length of protrusive and invasive structures into the collagen type I matrix (Fig. 2g-i). As a control, we have performed dasatinib treatment in P-cadherin low breast cancer cells (MCF-7/AZ.mock and BT20 siCtr) and, as expected, the impact of the dasatinib treatment is very modest, since the pathway is not as activated as in P-cadherin overexpressing cells (Additional file 1: Figure S2A-C) [22].

Altogether, the results support that SRC activity suppression by dasatinib is able to prevent the functional effects induced by P-cadherin overexpression in breast cancer cells.

### Dasatinib treatment promotes the recovery of cell-cell adhesion function by stabilizing E-cadherin/p120ctn complex at the cell membrane

**Fig. 3:**
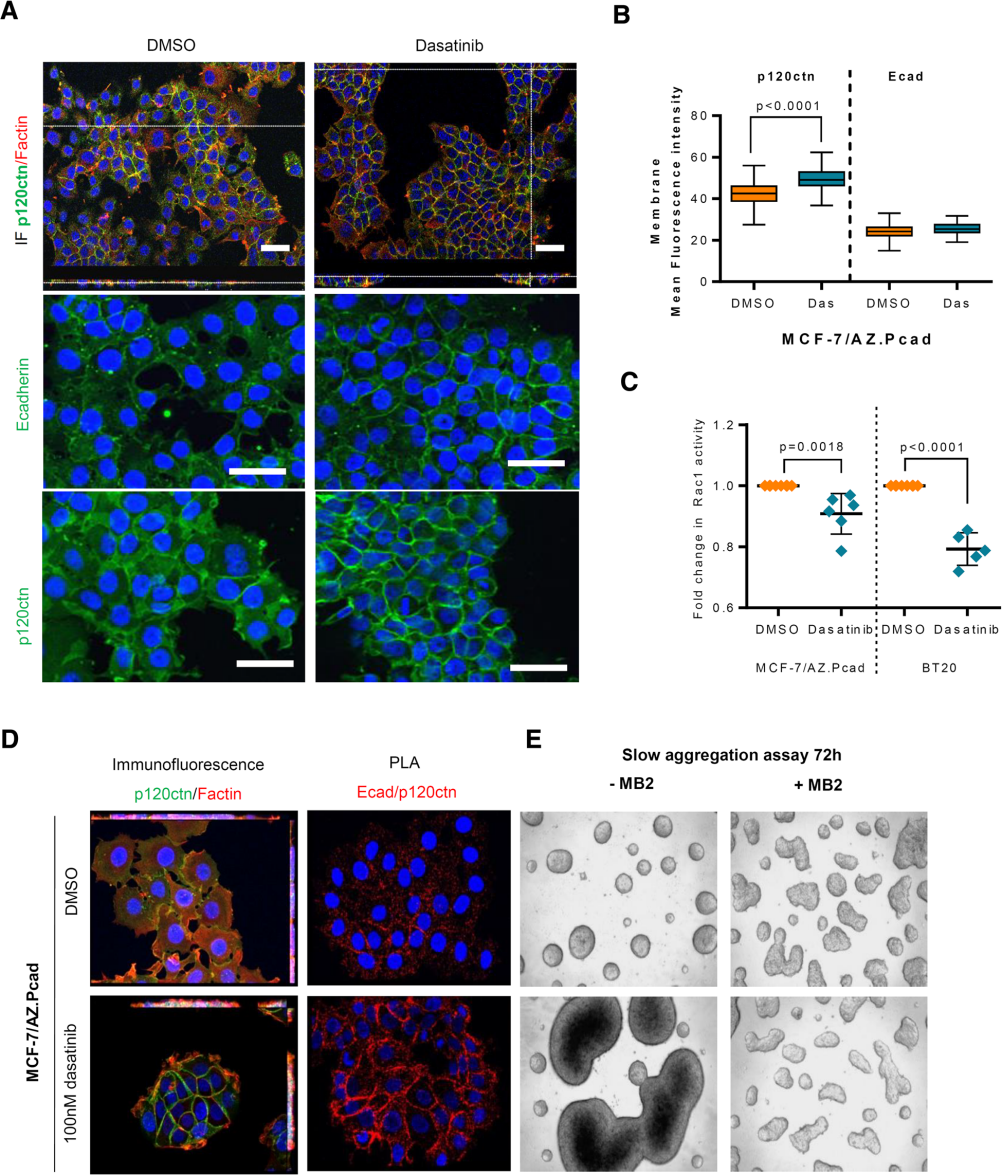
Dasatinib treatment promotes the recovery of E-cadherin function with stabilization of the E-cad/p120ctn complex to the cell membrane. **a** Dual Immunofluorescence for anti-p120ctn antibody (green), F-actin (red), E-cadherin (green) and DAPI (blue) in MCF-7/AZ.Pcad cells after 100 nM dasatinib treatment for 48 h. Scale bar = 50 μm. **b** Internuclear profile for p120ctn and E-cadherin, obtained through the computational analysis of confocal immunofluorescent images. P-values indicate significant differences observed in the membrane mean fluorescent intensity for p120ctn between MCF-7/AZ.Pcad treated with DMSO versus 100 nM dasatinib, 48 h. **c** Quantification of the active Rac1-GTP, measured by a G-Lisa assay, was performed to measure Rac1 activity in P-cadherin overexpressing cells (MCF-7/AZ.Pcad and BT20) treated with DMSO and dasatinib. **d** Proximity ligation assay for E-cadherin and p120ctn, for MCF-7/AZ.Pcad treated with DMSO versus 100 nM dasatinib during 48 h. **e** 72 h slow aggregation assay images for dasatinib treated MCF-7/AZ.Pcad cells with inhibition of E-cadherin function by MB2 antibody. The images shown are representative ones. P-values are indicated in the figure and considered statistically significant when < 0.05

We have previously shown that P-cadherin overexpression in an E-cadherin wild-type context weakens cell-cell adhesion by disrupting the E-cadherin/catenins complex [11]. Taking this into account, we evaluated the effect of dasatinib at this molecular level. As observed in Fig. 3a, dasatinib promoted an increase in membrane expression of p120ctn, as observed by its internuclear profile (Fig. 3b), which was associated with a decrease in the downstream activation of Rac 1 (Fig. 3c). Once again, E-cadherin expression and localization was not altered (Fig. 3b), although dasatinib treatment promoted a significant recover of the E-cadherin/p120ctn complex, mainly at the cell membrane (Fig. 3d and Additional file 1: Figure S3A and B). Both results show that activation of SRC signaling interferes with E-cadherin/p120ctn complex in agreement with the disruption of cell-cell adhesion. These results were further validated using a selective SRC inhibitor (PP2) and by SRC silencing using siRNA (Additional file 1: Figure S4). We confirmed, by slow aggregation assays, that dasatinib promoted cell-cell adhesion of P-cadherin overexpressing cells by rescuing E-cadherin function, since the treatment with MB2 antibody (that blocks E-cadherin function) completely abolished cell-cell adhesion capacity induced by dasatinib (Fig. 3e and Additional file 1: Figure S2B).

### Dasatinib affects in vivo tumorigenic and metastatic capacity of P-cadherin overexpressing breast cancer cells

**Fig. 4:**
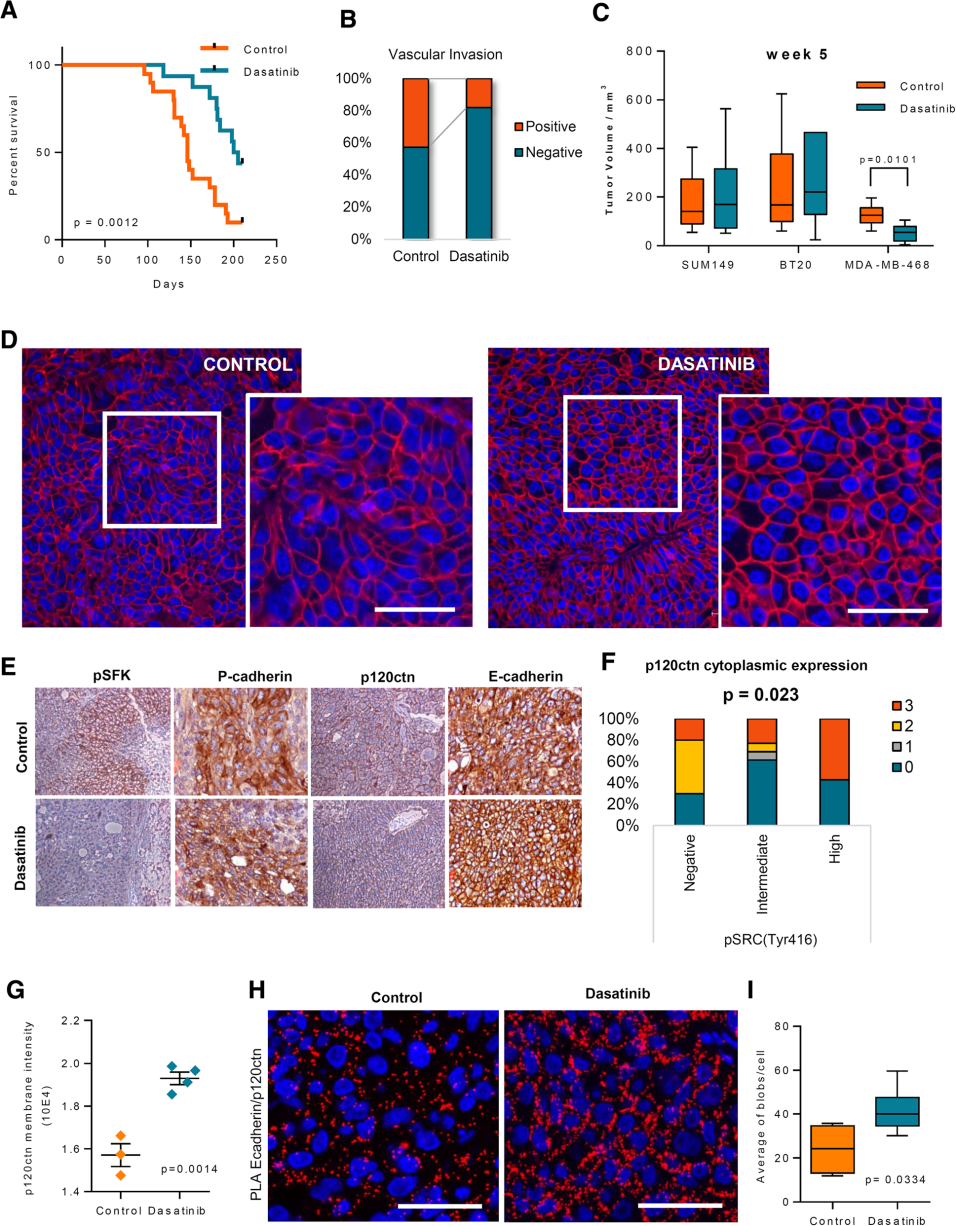
Dasatinib treatment affects the in vivo tumorigenic and metastatic capacity of P-cadherin overexpressing tumors. **a** Kaplan-Meyer survival curve for the overall survival of mice treated with DMSO and dasatinib 10 mg/kg, for a maximum period of 210 days. P-value was calculated using a log-rank test to assess significant differences for mice overall survival. **b** Percentage of tumours with vascular invasion, observed by HE staining. Presence or absence of clusters of tumour cells inside blood vessels surrounding the primary tumour was quantified in control versus dasatinib treated mice (10 mg/kg). **c** Tumour volume measured at week 5, post inoculation of cancer cells, from control and dasatinib treated groups, in all BCC models (SUM149, BT20 and MDA-MB-468). **d** Immunofluorescence for p120ctn (red) and DAPI (blue in the primary tumours from control and dasatinib treated animals. Scale bar = 50 μm. **e** Immunohistochemistry for p120ctn and pSRC(Tyr416) in the primary tumours from control and dasatinib treated animals. The images shown are representative ones. **f** Quantification of the p120ctn cytoplasmic intensity expression in primary tumours from control and dasatinib treated animals. **g** Quantification of the p120ctn membrane intensity. Each dot represents the mean of p120ctn membrane intensity of 100 pairs of cells from a single biological replicate. Student’s t-tests were used to determine statistically significant differences, and P-values are indicated in the figure. Scale bar = 50 μm. **h** Proximity ligation assay for E-cadherin and p120ctn, from tumours recovered from different mice from control and dasatinib treated groups. **i** Box-plot for the average number of blobs/cells, quantified using the images of the proximity ligation assay for E-cadherin and p120ctn, between tumours from control and dasatinib treated mice. Student’s t-tests were used to determine statistically significant differences, and the P-value are indicated in the figure. Scale bar = 50 μm

In order to evaluate the inhibitory tumorigenic potential of dasatinib, we used pre-clinical mouse models. We developed an orthotopic model and protocol to study the effect of dasatinib treatment in tumour evolution and recurrence (Additional file 1: Figure S5). Three distinct P-cadherin overexpressing cell lines were analysed: SUM-149, BT20 and MDA-MB-468. Dasatinib treated mice showed an improvement in the wellbeing, as measured by the % of weight gain (data not shown). Further, dasatinib treated animals had a significant increase in overall survival time (Fig. 4a and Additional file 1: Figure S6A), with a tendency to show a decrease in vascular invasion, as observed in the histology from removed primary tumors (Fig. 4b). Interestingly, a significant decrease in tumour growth (Fig. 4c) was observed with MDA-MB-468 cells at week 5 post-inoculation, although the same is not observed for the BT20 and SUM149 BLBC cell model Molecularly, P-cadherin co-localizes with pSRC(Tyr416) expression in the tumours recovered from the mice (Additional file 1: Figure S6B). Importantly, modifications in the pattern of p120ctn expression and cell-cell organization were also observed in dasatinib treated tumours. A significant increase in membrane p120ctn staining (Fig. 4d-e) could be observed in these tumours, as well as a significant correlation was found between pSRC(Tyr416) staining and p120ctn cytoplasmic expression (Fig. 4f). As found in in vitro models, a recovery of the E-cadherin/p120ctn complex interaction to the cell membrane has also been found in tumours treated with dasatinib, as measured by PLA (Fig. 4g-i).

### CDH3 predicts sensitivity to dasatinib treatment in basal-like triple negative breast cancer

In view of all in vitro and in vivo data, we hypothesized that P-cadherin expression could be classified as a predictive biomarker to stratify BL-TNBC patients for dasatinib treatment.

**Fig. 5:**
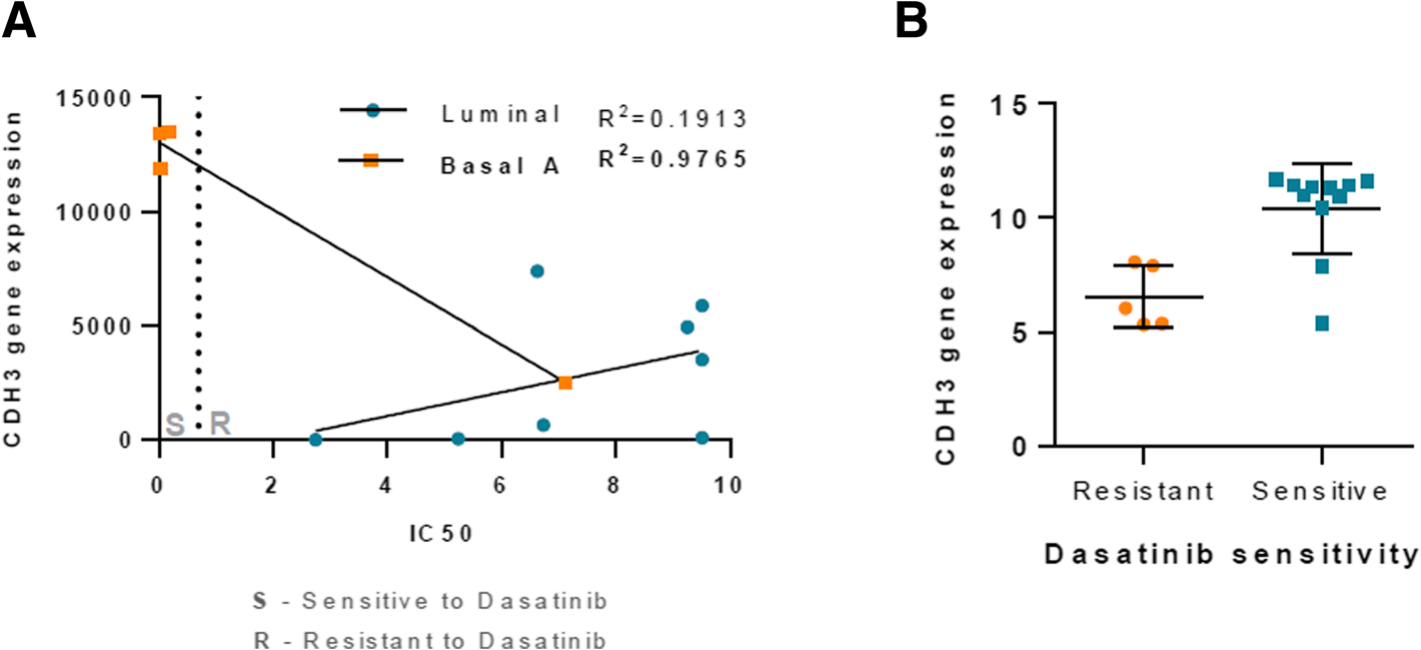
*CDH3* expression predicts sensitivity to dasatinib treatment. **a** Correlation between *CDH3* gene expression and IC50 values for dasatinib treatment in Luminal and Basal A BCC lines. The data was retrieved from a previously published gene expression profile using 23 breast cancer cell lines to identify genomic signatures highly correlated with in vitro sensitivity to dasatinib (GEO accession number: GSE6569). **b** *CDH3* gene expression values in dasatinib sensitive and resistant prostate cancer cells. The data was retrived using a dataset of baseline gene expression profiling of 16 prostatic cancer cell lines used to identify expression signatures highly correlated with in vitro sensitivity to dasatinib (GEO accession number: GSE9633 [25])

By the analysis of a previously published gene expression profile of a data set of BCC lines (GEO accession number: GSE6569) [23], we observed that *CDH3* expression correlates with the sensitivity/resistance of breast cancer cells to dasatinib [24]. We have found a statistically significant association between dasatinib sensitivity and increased *CDH3* expression in Basal A BCC (Fig. 5a). The same holds for a series of prostate cancer cell lines (GEO accession number: GSE9633) [25] (Fig. 5b).

## Discussion

P-cadherin is overexpressed in more than 50% of triple-negative breast cancer and is significantly associated with poor patient survival [7, 26, 27], showing a key role in some acquired cancer hallmarks, such as increased cell migration, invasion, as well as tumorigenic and metastatic capacity in breast cancer models [9, 11]. Actually, P-cadherin can be considered as a therapeutic target in breast cancer, with a particular interest in BLBC due to the lack of targeted therapies in this specific subgroup of patients.

Molecularly, P-cadherin in vitro effects are due to inhibition of the E-cadherin suppressive invasive function, by disruption of the E-cadherin/p120catenin complex at the cell membrane [11]. SRC activation is also described to have a crucial role in impairing E-cadherin function and p120ctn delocalization to the cytoplasm [17, 28], which points for a possible role of SRC activation in P-cadherin mediated signalling.

Considering all that, our first aim was to show the link between P-cadherin and SRC activation. Using both gene expression profiles, in vitro and patient data, we have demonstrated a clear association between P-cadherin/*CDH3* expression and SRC signature. Actually, previous publications have already demonstrated, in normal and in distinct cancer cell models, that P-cadherin expression activates SRC through its tyrosine phosphorylation, being considered one of its main signalling pathways to induce invasive and stem cell behaviour [13, 18, 22, 29].

SRC inhibitors were shown to be the most successful and promising drugs for TNBC treatment [30, 31]. Specifically, dasatinib (BMS-354825) was identified as a highly potent inhibitor of SFK, showing anti-proliferative, anti-migratory and anti-invasive activity in solid tumors [25]. Nevertheless, clinical trials involving dasatinib for breast cancer treatment showed limited activity, even in BL-TNBC subgroup [23, 32, 33]. These negative results could be most probably due to the lack of previous selection of the patients that would in fact benefit from this therapy, as well as due to the high heterogeneity of TNBC. Therefore, in our opinion, it is imperative to identify predictive biomarkers linked to SRC signalling pathway that may constitute a tool for therapeutic eligibility to dasatinib.

Taking these previous observations into account, and knowing that the invasive P-cadherin cells have activation of the SRC pathway [34–37], we tested the functional effect of dasatinib in P-cadherin overexpressing tumour cells, in order to evaluate the impact of this adhesion molecule as a therapeutic biomarker in BLBC.

**Fig. 6:**
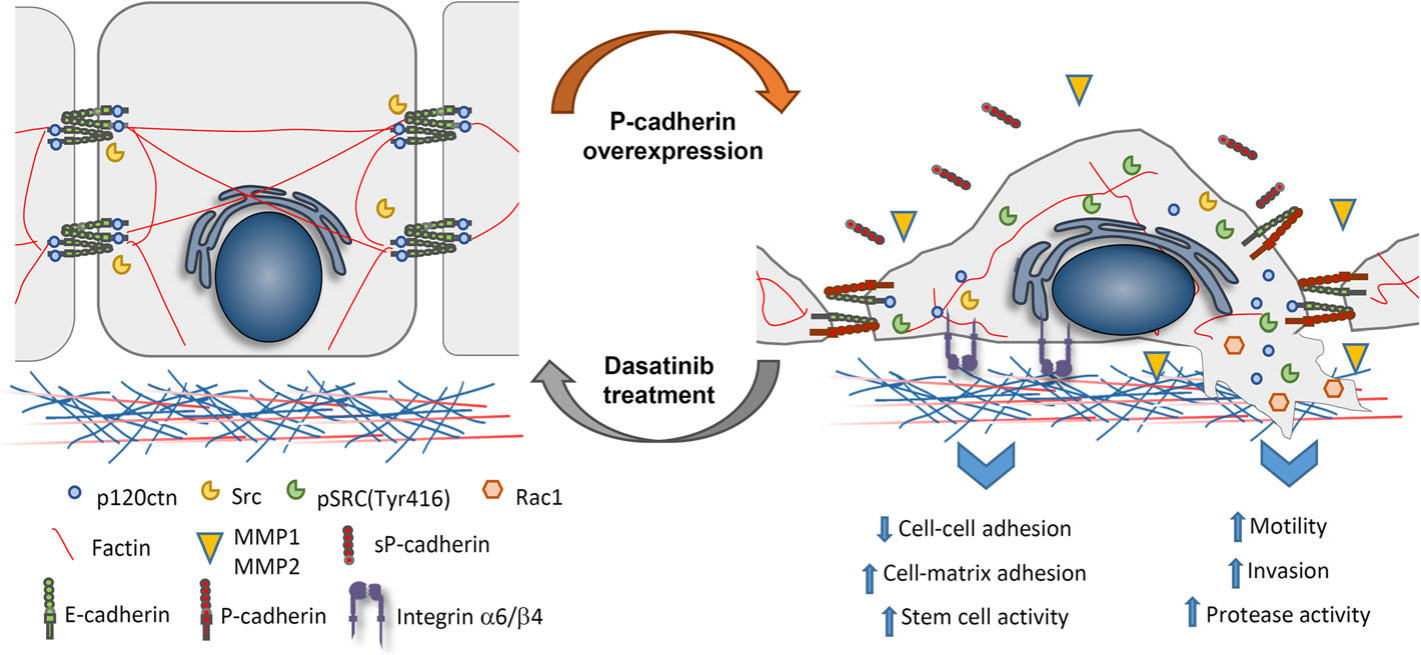
Schematic representation of the proposed model. P-cadherin-overexpressing cells significantly alter their biomechanical properties, presenting a decreased cellular height, cell stiffness and cell-cell adhesion strength. The activation of a mechanotransduction signaling initiated by P-cadherin overexpression, promotes an activation of SRC, with a concomitant delocalization of p120ctn to the cytoplasm, disrupting the E-cadherin/p120ctn complex, and activating the Rac1 small GTPase. Consistently, P-cadherin-overexpressing cells show a significantly higher number of protrusions, as well as increased protrusion’s length, increased cell migration, invasion, protease activity and self-renew potential. Molecularly, the expression of this basal marker affects: 1) cell-cell adhesion, through inhibition of the E-cadherin suppressive invasive function; 2) cell-ECM interaction, activating the heterodimer α6β4 integrin and 3) cell-ECM crosstalk, by regulating the activity of MMP1/MMP2 metalloproteases. Interestingly, inhibiting P-cadherin downstream SRC activation, using the FDA approved drug dasatinib, lead to a significant decrease in both morphological, biomechanical and functional properties induced by P-cadherin expression. In conclusion, the results anticipate an important role for P-cadherin in the mechanotransduction signaling in BCCs, which could be further inhibited by dasatinib

Our results showed that dasatinib inhibits P-cadherin/SRC signalling significantly preventing the in vitro cellular effects induced by P-cadherin expression, leading to a significant decrease in cell migration and invasion, proteases secretion and mammosphere-forming efficiency. Additionally, dasatinib treatment promoted a recovery of an epithelial-like phenotype and induced the membrane stabilization of the E-cadherin/p120ctn complex, with a significant increase in cell-cell adhesion capacity. This result was further confirmed by using a SRC selective inhibitor and a siRNA specific for SRC. This data strengthens previous studies, showing that inhibition of this signaling pathway increases cell-cell adhesion in vivo [38], promoting an epithelial-like morphology and suppressing epithelial tumor cell migration. In this work, we demonstrated, for the first time, that SRC inhibition, which is downstream activated by P-cadherin, recovers the E-cadherin-p120ctn stability and function, which is crucial for cell-cell adhesion and suppression of tumorigenesis. Remarkably, P-cadherin overexpressing cells and tumors treated with dasatinib show a clear shift of p120ctn expression from the cytoplasm to the membrane and a recovery of E-cadherin/p120ctn complex interaction at the cell membrane. Phenotypically, and according to in vitro results, dasatinib treated mice showed tumors with an epithelial-like phenotype. Both observations support that dasatinib treatment, by inhibition of SRC, is able to inhibit P-cadherin induced signaling, promoting a non-invasive epithelial-like phenotype (Fig. 6). Additionally, CDH3 expression was able to predict sensitivity/resistance to dasatinib treatment, both in breast cancer and in prostate cancer cell models. Therapeutically, our observations indicate that dasatinib is likely to be an interesting option to treat P-cadherin overexpressing tumours.

Although the effects on tumour growth were not replicated in the different BLBC cell models used, consistent results were observed in disease progression. Actually, using mice xenografts, we were able to validate the dasatinib effect on decreasing tumour invasion of P-cadherin overexpressing cells, as well as in the improvement of mice wellbeing and overall survival of dasatinib treated mice. This result is in accordance with the in vitro data, since we do not observe differences in the cell proliferation of dasatinib treated cells, but instead we see a significant impact on the aggressive biological behavior of P-cadherin overexpressing cells. Similar results were obtained in an in vivo pancreatic cancer model, where dasatinib delayed tumor onset and increased overall survival [35].

## Conclusions

In conclusion, our work shows for the first time that dasatinib counteracts P-cadherin mediated signalling and functional effects, suggesting this protein as a possible biomarker to predict sensitivity/resistance to SRC-directed therapy. Molecular guiding and selection of patients will provide the correct tools to treat P-cadherin overexpressing and highly aggressive breast carcinomas.

## Additional file

Additional file 1: Supplementary Table S1 and Figures S1 - S6. (PDF 1080 kb)

## Abbreviations

BLBC: Basal-like breast cancer
FAK: Focal adhesion kinase
FDA: USA Food and Drug Administration
p120ctn: p120 catenin
SRC: Src family kinase
