# Supplementary material for: SRC inhibition prevents P-cadherin mediated signaling and function in basal-like breast cancer cells
Source: Cell Commun Signal. 2018 Nov 7;16:75. doi: 10.1186/s12964-018-0286-2 (PMC6223051; doi:10.1186/s12964-018-0286-2)
Supplement: Supplementary file 1 — Supplementary Table S1 and Figures S1 - S6. (PDF 1080 kb) [file 12964_2018_286_MOESM1_ESM.pdf]

Addtional file 1

Protein	Clone	Species	Western blot dilution		Immunofluorescence		Immunohistochemistry	
			dilution	Incubation time	dilution	Incubation time	dilution	Incubation time
E-cadherin	HECD-1	Mouse	1:1000	ON, 4ºC	-	-	-	-
E-cadherin	24E10	Rabbit	-	-	1:100	1h, RT	1:100	1h, RT
p120ctn	98	Mouse	1:1000	ON, 4ºC	1:100	1h, RT	1:100	1h, RT
P-cadherin	56	Mouse	1:500	ON, 4ºC	-	-	1:100	1h, RT
P-cadherin	C13F9	Rabbit	-	-	1:50	ON, 4ºC	-	-
β-actin	I19	Goat	1:1000	ON, 4ºC	-	-	-	-
Total Src	L4A1	Rabbit	1:1000	ON, 4ºC	1:100	ON, 4ºC	1:40	ON, 4ºC
pSFK(Tyr416)	100F2	Rabbit	1:1000	ON, 4ºC	1:50	ON, 4ºC	1:40	ON, 4ºC

Table S1. Detailed description of the experimental conditions for the antibodies used.

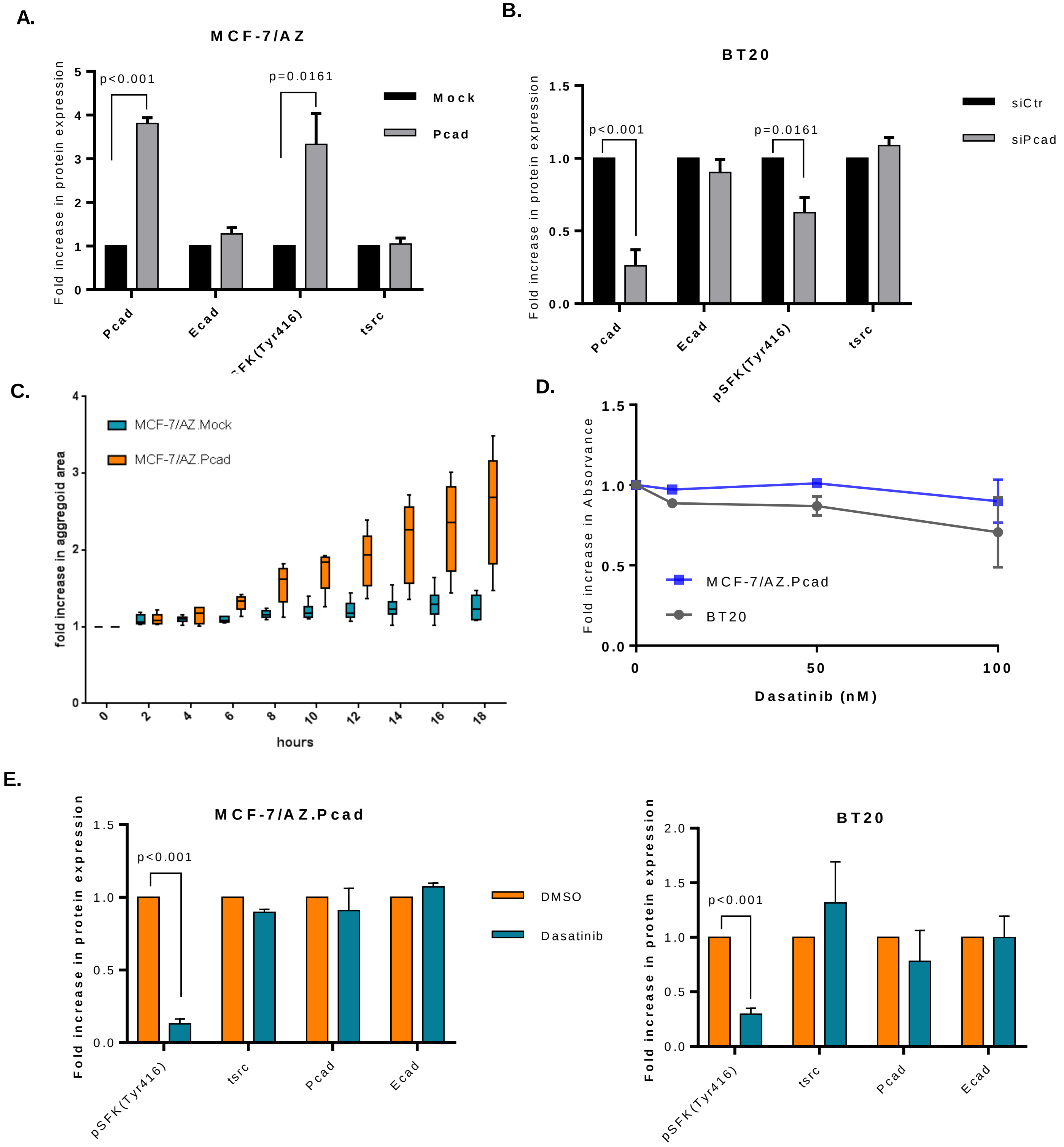

Figure S1. A) Fold change in protein expression, detected by western blot, for P-cadherin, E-cadherin, pSFK(Tyr416) and total Src in MCF-7/AZ.Mock versus MCF-7/AZ.Pcad cells. B) Fold change in protein expression, detected by western blot, for P-cadherin, E-cadherin, pSFK(Tyr416) and total Src in BT20 siCtr versus BT20 siPcad cells. C) Fold increase in aggregoid area, reflecting invasion capacity of cancer cells in 3D collagen gel; D) Fold change in absorvance, using the MTT assay, reflecting the metabolic state of BCC, for both MCF-7/AZ.Pcad and BT20 models, treated with different concentrations of dasatinib, for 24h. E) Fold change in protein expression, detected by western blot, for pSFK(Tyr416), total Src, P-cadherin and E-cadherin, in P-cadherin overexpressing cells (MCF-7/AZ.Pcad and BT20) treated with 100nM of dasatinib for 48h. All Western blot analysis was performed by quantifying the band density, using Quantity one. The levels of our protein of interest were normalized with the respective loading control (β-actin). Student's t-tests were used to determine statistically significant differences, and the P-value are indicated in the figure.

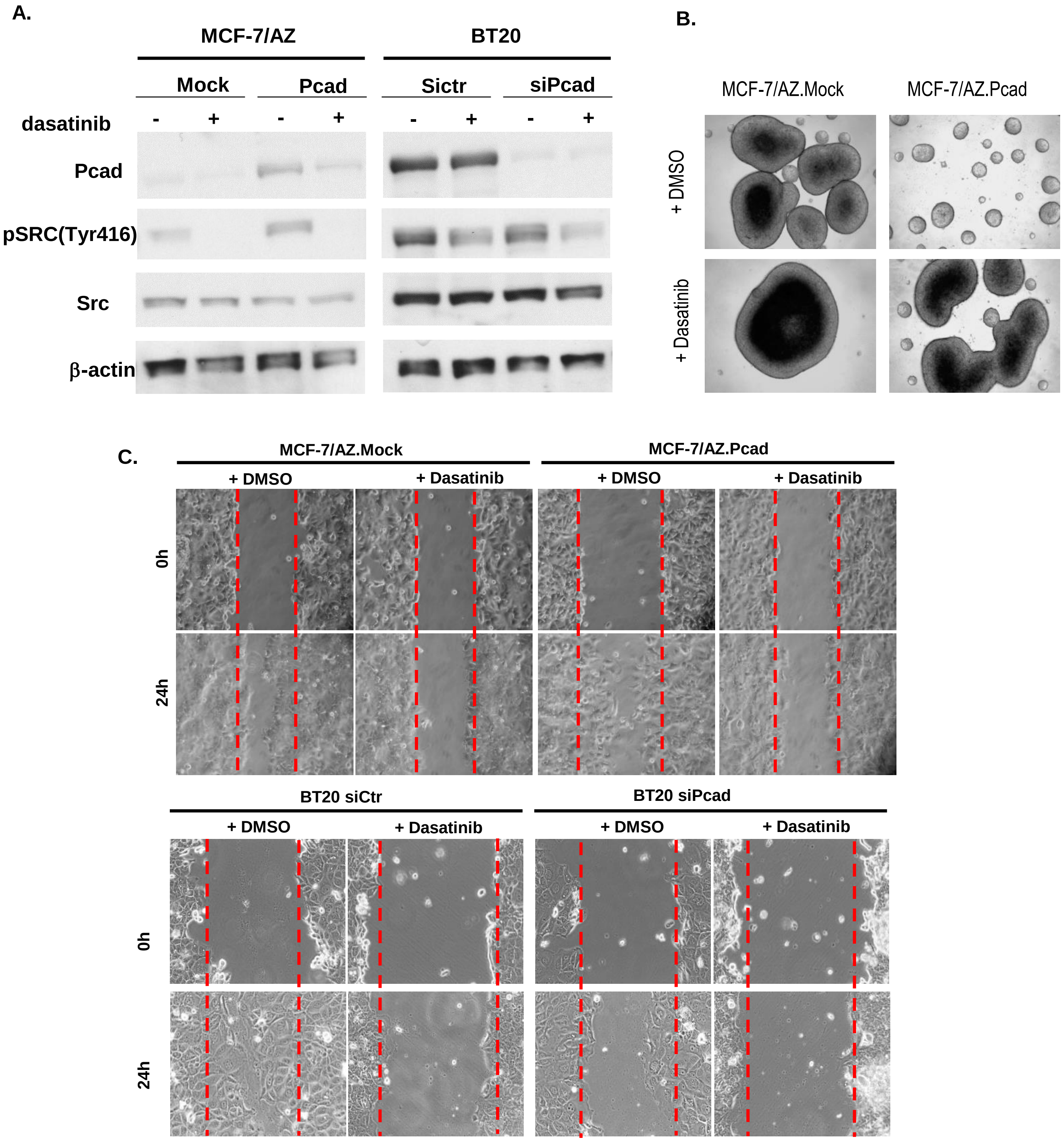

Figure S2. A) Western blot, for P-cadherin, E-cadherin, pSRC(Tyr416) and total Src in MCF-7/AZ. And BT20 models with dasatinib treatment. B) 72h slow aggregation assay images for Dasatinib treated MCF-7/AZ cell model; C) Representative experiment from a wound healing migration assay, in both MCF-7/AZ and BT20 model, treated with 100nM of Dasatinib or DMSO for 24h. C

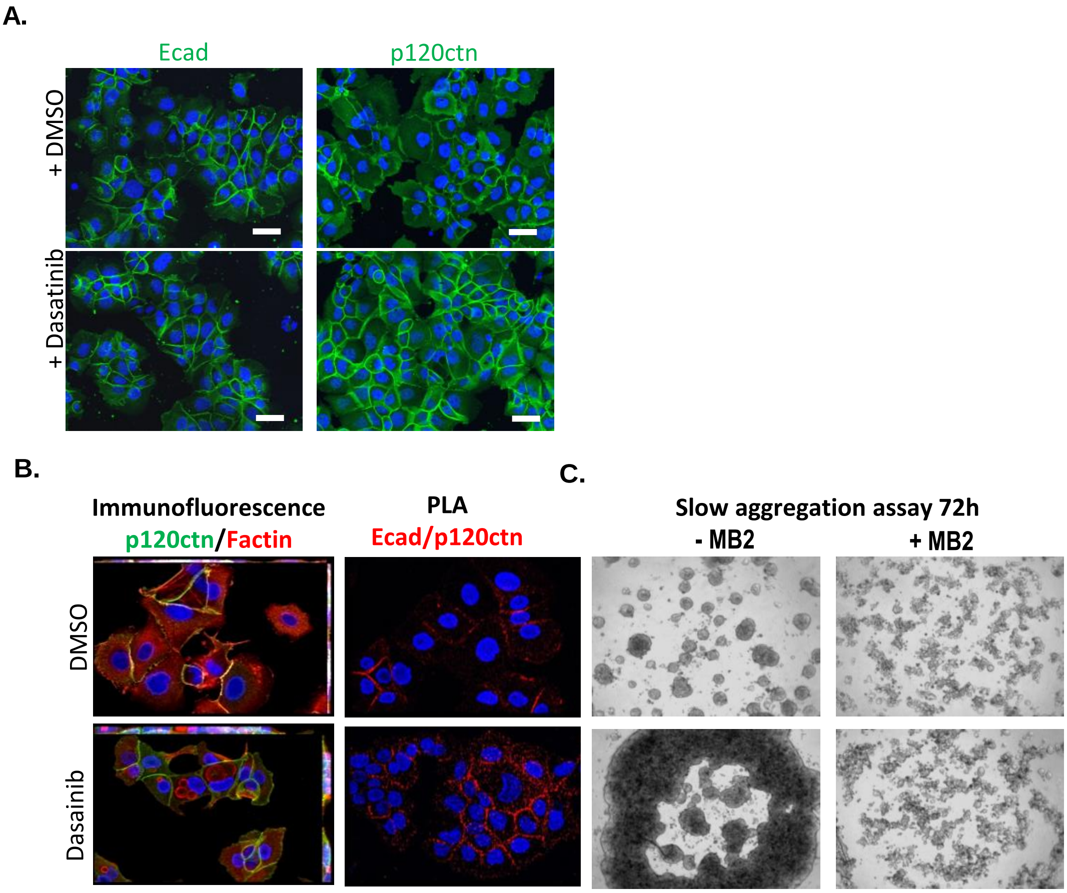

Figure S3. A) Immunofluorescence for anti-p120ctn antibody (green), E-cadherin (green) and DAPI (blue) in BT20 cells after 100nM dasatinib treatment for 48h. B) Dual Immunofluorescence for anti-p120ctn antibody (green), F-actin (red) and DAPI (blue). Proximity ligation assay for E-cadherin and p120ctn, for BT20 treated with DMSO versus 100nM dasatinib during 48h. The images shown are representative ones. A minimum of three independent biological replicates were performed. C) 72h slow aggregation assay images for Dasatinib treated BT20 cells with inhibition of E-cadherin function by MB2 antibody. The images shown are representative ones. Scale bar = 50μm.

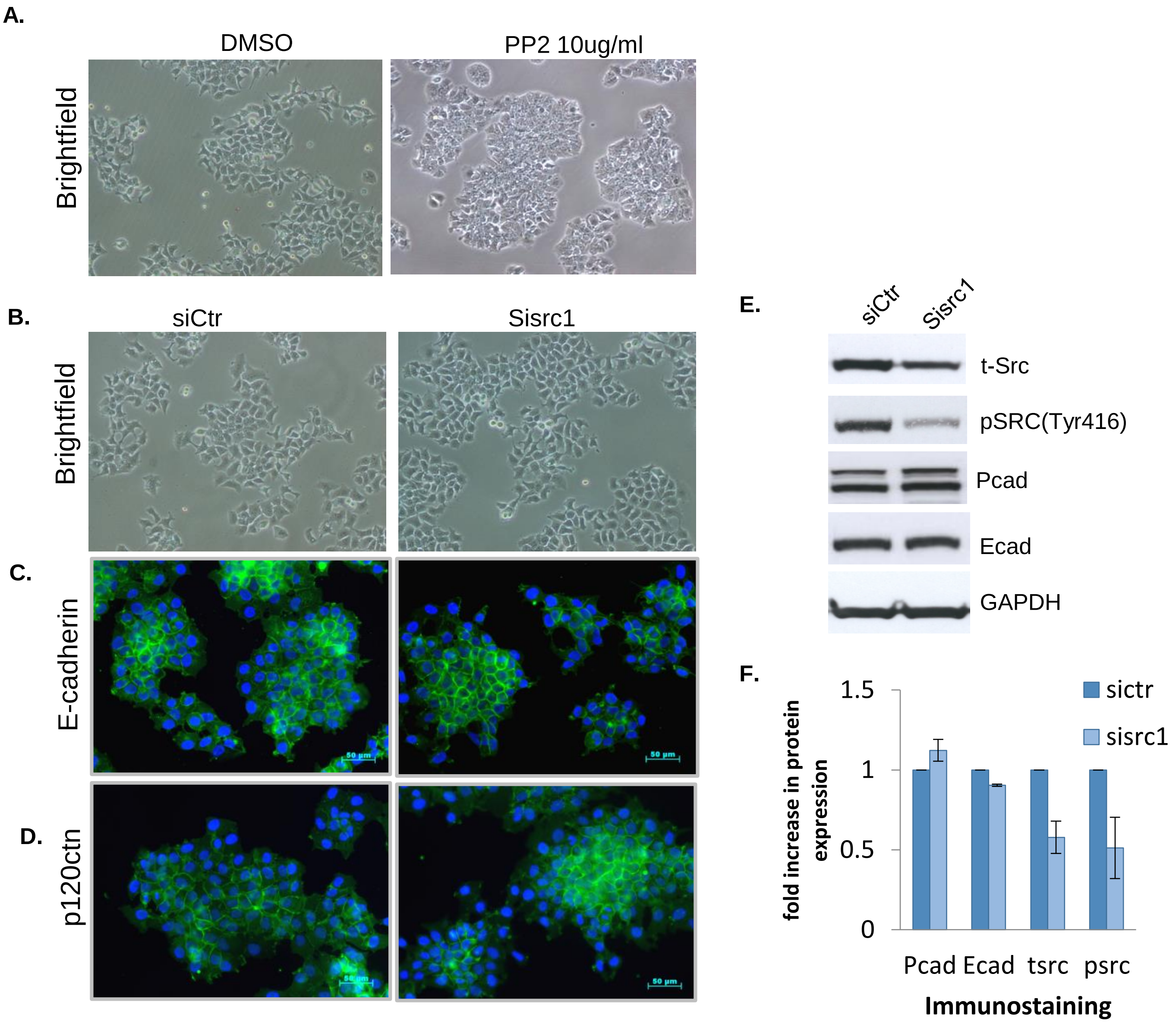

Figure S4. Brightfield images to evaluate cell morphology for MCF-7/AZ.Pcad cells treated with PP2 (A) and silenced for src (B). Immunofluorescence for anti-E-cadherin antibody (green) (C), p120ctn (green) (D) and DAPI (blue) in MCF-7/AZ.Pcad cells 48h after silencing Src with siRNA. Western blot representative images for total src, pSRC(Tyr416), P-cadherin and E-cadherin (E). The images shown are representative ones. A minimum of three independent biological replicates were performed and quantified by Quantity One (F). Scale bar = 50μm.

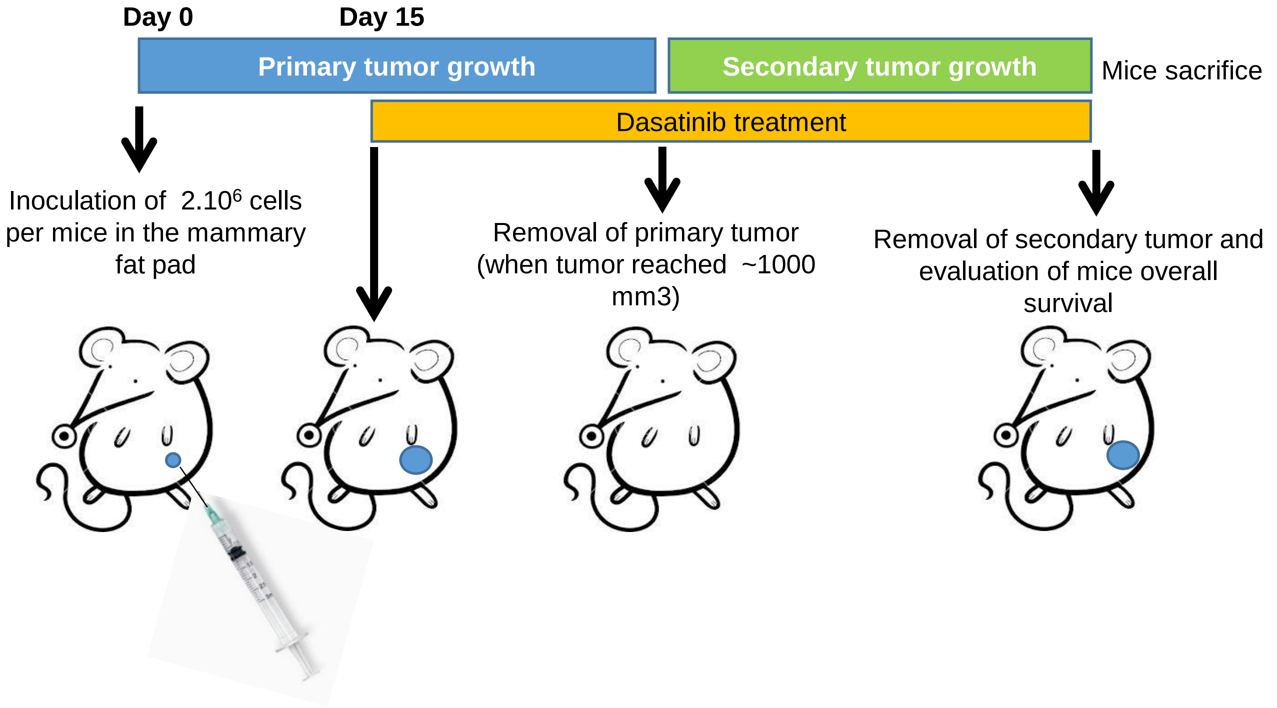

Figure S5. Schematic representation of the experimental design performed in the pre-clinical *in vivo* study, following the model of tumor formation. The experiments consisted on the orthotopic injection in the mammary fat pad of female mice, with 6-8 weeks of age, of 2x10^6 cells from each P-cadherin-overexpressing breast cancer models (SUM149PT, n=17; BT20, n=15; MDA-MB-468, n=13) using a 25G needle. When the induced primary tumours reached a mean volume of 100 mm^3, mice were randomized into two groups, and started the oral daily treatment with Dasatinib, whereas the others were treated just with the drug vehicle, until the end of the experiment (maximum 210 days). Every week, daily doses of Dasatinib were prepared, by dilution in citrate buffer 80 mM (pH=3.1), and mice were treated with a final concentration of 10 mg/kg. Tumors were surgically removed when an average volume of 1000 mm^3 was reached, and mice were maintained to evaluate the impact of Dasatinib in mice overall survival, for a maximum of 210 days, unless they showed any signs of disease progression.

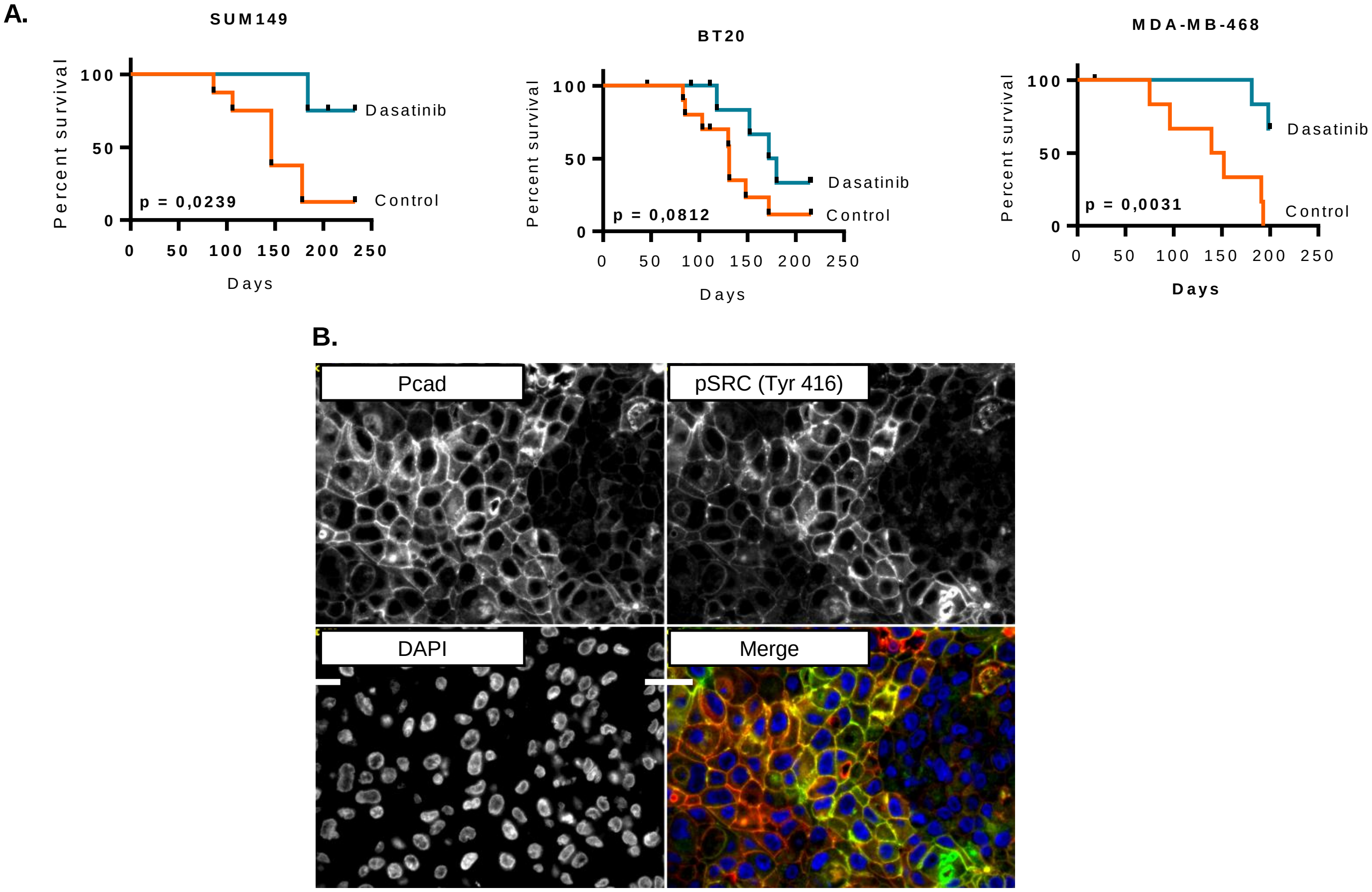

Figure S6. A) Kaplan-Meyer survival curve for the overall survival of mice treated with DMSO and Dasatinib 10mg/kg, for a maximum period of 210 days, in the different BCC models (SUM149 PT, BT20 and MDA-MB-468). P-values were calculated using a log-rank test to assess significant differences for mice overall survival. P-values <0.05 were considered as statistically significant. B) Dual Immunofluorescence for anti-Pcadherin antibody (red), pSFK(Tyr416)(green) and DAPI (blue) in primary tumors. Scale bar = 50µm. The images shown are representative ones
